# The Relationship between Psychological Stress and Anthropometric, Biological Outcomes: A Systematic Review

**DOI:** 10.3390/medicina60081253

**Published:** 2024-08-01

**Authors:** Joanna Rog, Katarzyna Nowak, Zuzanna Wingralek

**Affiliations:** 1 Laboratory of Human Metabolism Research, Department of Dietetics, Institute of Human Nutrition Sciences, Warsaw University of Life Sciences (WULS-SGGW), Nowoursynowska 66 Str., 02-787 Warsaw, Poland; 2 1st Department of Psychiatry, Psychotherapy and Early Intervention, Medical University of Lublin, 20-950 Lublin, Poland; katarzyna.nowak235@gmail.com (K.N.); z.wingralek@gmail.com (Z.W.)

**Keywords:** distress, body compound, body fat, metabolism, cortisol, psychological stress

## Abstract

*Background and Objectives*: Challenges and threats to global security and the growing demands of today’s society lead to significantly increased exposure to stress. Stress can negatively affect numerous physiological processes, including metabolic changes. An unhealthy lifestyle might intensify this disruption. The aim of the systematic review was to establish the effect of psychological stress on metabolic and anthropometric factors in healthy individuals. *Materials and Methods:* The study was conducted according to the PRISMA guidelines; and the risk of bias (ROB) assessment was based on the Newcastle–Ottawa Scale (NOS). A literature search of the MEDLINE/PubMed database was conducted using specific search terms. *Results:* We identified 32 articles meeting the inclusion criteria for the review with the different experimental designs and aims. Most of the papers were at high ROB. The included studies were conducted in groups of adults and children/teenagers. The most-often-applied tool to measure stress severity was the Perceived Stress Scale (PSS). Twenty-two studies analyzed the connection between stress and body composition, and bioimpedance analysis (BIA) was the most often used method. For biological parameters, the most frequently analyzed was cortisol (n = 9). The other examined factors included glucose, insulin, parameters related to food intake regulation, carbohydrates, lipid metabolism, inflammation, and oxidative stress. The included studies were incompliance in relation to the assessment method and type of assessed biological fluids. *Conclusions:* The vast majority of studies do not support the effect of chronic distress on anthropometric measurements and biological markers levels. However, many of them suggest adverse, synergistic effects of unhealthy lifestyle patterns and the stress on the examined variables. Further experiments should implement a similar and repeatable methodology.

## 1. Introduction

According to The American Institute of Stress, society is facing a mental health crisis that could yield serious negative consequences for years to come, and the global average number of stressed people is 35% [1]. Stress is the body’s non-specific response to any demand for change or, in other words, physical, mental, or emotional strain or tension. Acute experience of this phenomenon is suitable for people to perform themselves and motivate in a specific context [2]. However, prolonged psychological stress (or distress) leads to adverse health-related outcomes later in life. The growing demands of today’s society regarding employment or socioeconomic status, time pressure on the work–life balance, and high academic expectations from parents result in being overwhelmed and burning out [3,4]. 

Considering the challenges and threats to global security (COVID-19, Russian invasion of Ukraine) after 2017, stress levels have significantly increased. Nearly 35% of individuals around the world experienced significant stress during the coronavirus pandemic, and according to studies conducted in 17 European countries, a decline in well-being on the day of the Russian invasion was observed [5,6]. Psychological distress is a suspected cause of many chronic diseases, referred to not only as mental health problems. The stress was related to increased risk of diabetes mellitus, chronic obstructive pulmonary disease, autoimmune and cardiovascular diseases, and all-cause and cardiovascular disease-specific mortality [7,8,9,10]. The mechanisms underlying stress-induced development of long-lasting illnesses have not been fully elucidated. Psychological distress could negatively affect lifestyle, resulting in poor diet, physical inactivity, insomnia, smoking, and alcohol intake. Unhealthy behaviors lead to obesity development, affecting increased risk for many non-communicable diseases. A recent report published by the World Health Organization (WHO) estimates that overweight and obese affects almost 60% of adults [11]. 

Stress can negatively affect numerous psychological processes, disturbing the human body’s homeostasis. The model studies confirm the role of the feeling of strain in intestinal barrier dysfunction, microbiota dysbiosis, and systemic inflammation [12,13]. Another proposed phenomenon linking psychological stress to chronic disease is metabolic disturbances concerning various metabolic pathways such as amino acids, lipids, carbohydrates, adipokines, and energy metabolism [14,15,16,17]. 

The role of psychological stress on metabolic and anthropometric parameters in healthy individuals has yet to be well established. Despite much data from model studies and reviews focusing on the direct development of disease due to stress, a comprehensive analysis of the more detailed effects of distress on the human body still needs to be provided. The systematic review aims to establish the impact of psychological stress on metabolic and anthropometric measures/factors in healthy individuals.

## 2. Materials and Methods

### 2.1. Design

To ensure the comprehensive and transparent reporting of this review, the “PRISMA statement for reporting systematic reviews and meta-analyses of studies that evaluate health care interventions: explanation and elaboration” was used [18]. The study was registered in Open Science Framework (Identifier: DOI 10.17605/OSF.IO/SZ48M).

### 2.2. Search Strategy

A literature search of the MEDLINE/PubMed database was conducted for articles published in the English language from database inception to 27 December 2022 with two updates (13 February and 25 May). Additional studies were identified by hand-searching the references of the included studies and of found systematic reviews and meta-analyses in the database. Search terms included: psychological stress, metabolic disruptions, metabolome, metabolism, body fat, and body composition. We typed the following phrase in the query box: ((((((((psychological stress) AND (metabolic disruptions)) OR (psychological stress)) AND (metabolome)) OR (psychological stress)) AND (metabolism) OR (psychological stress)) AND (body fat) OR (psychological stress)) AND (body composition)). 

### 2.3. Inclusion/Exclusion Criteria

Only human studies that were original papers written correctly in English were included. No publication date or publication status restrictions were imposed. Participants of any age without any reported disease were considered. The restriction was not imposed on the body mass index (BMI) value or the presence of chronic disease in some individuals (however, we did not include clinical control studies in which disease or metabolic disruption was a differentiating factor or inclusion criterium). This review had no limitation on the type of study or the number of participants. The data on physiological stress and its relation to metabolic and anthropometric outcomes were collected without restriction. We analyzed only studies which examined body composition using a recognized method—for example, bioimpedance analysis (BIA) and dual-energy X-ray absorptiometry (DXA) (excluded BMI examination, which has limited accuracy/reliability to determine body fat). No principal summary measures were established due to the issue’s complexity, but only studies where stress was assessed by recognized methods (validated scales) were included.

### 2.4. Procedure of Data Extraction

**Figure 1:**
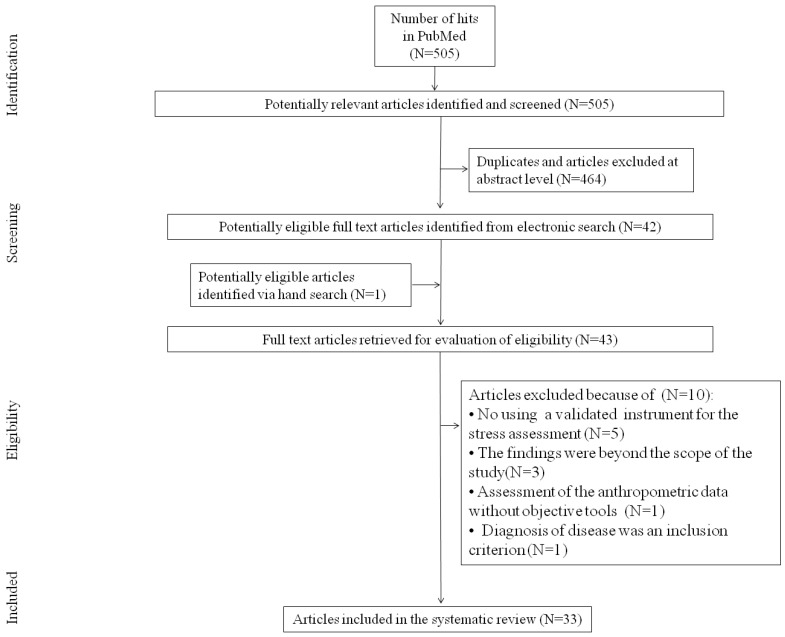
The methodological flow diagram of the systematic review.

Data were extracted and summarized from potentially relevant studies by two authors (JR and ZW) using predefined data fields, including study quality indicators. We used a 3-stage procedure to extract studies: (1) the verification of studies by title; (2) verification of included in the 1st step studies based on the abstract; (3) the final eligibility assessment was conducted by revising the full texts of potentially eligible studies. The procedures of identification, screening, assessment of eligibility, and inclusion are depicted in Figure 1. The following information was extracted from each included study: general knowledge of the study (author, type of study), country, aims, participants (number, gender, age, BMI, inclusion criteria, exclusion criteria, outcomes, methods of assessments, findings). 

### 2.5. Risk of Bias

According to the Cochrane guidelines [19], the methodological quality of the individual studies included in the review was assessed by the Newcastle–Ottawa Scale (NOS) [20]. Based on the NOS-obtained score, the results were interpreted by assignment to the following categories:

- Very high risk of bias (0–3 points)
- High risk of bias (4–6 points)
- Low risk of bias (7–9 points). Risk of bias (ROB) of individual studies was conducted by one of the authors (JR).

## 3. Results

In total, 505 unique articles were identified (123 abstracts were reviewed, 42 full texts of studies were assessed), and the finally selected 33 originated in the United States (n = 13) [21,22,23,24,25,26,27,28,29,30,31,32,33], Belgium (n = 4) [34,35,36,37], Canada (n = 3) [38,39,40], Greece (n = 2) [36,41], Switzerland (n = 2) [42,43], Finland (n = 2) [44,45], Netherlands (n = 1) [46], India (n = 1) [47], Brazil (n = 1) [48], Korea (n = 1) [49], Australia (n = 1) [50], Sweden (n = 1), Austria (n = 1), Hungary (n = 1), Spain (n = 1) [36], Mexico (n = 1) [51], United Kingdom (n = 1) [52], and Japan (n = 1) [53]. The papers were published between 2003 and 2023. The accumulated research studies observed different experimental designs and aims, as shown in Table 1.

The characteristics of the study participants are presented in Supplementary Table S2. Most studies included only adult participants (n = 16) [21,22,23,24,26,27,29,31,38,41,44,45,47,48,49,52]. Eleven experiments included children and teenagers [25,28,30,32,34,35,36,37,42,43,50], and 5 included pregnant women and their children to assess prenatal exposure to distress [33,39,40,46,51]. In 14 studies, the presence of chronic diseases (inconsistent in studies) [21,24,25,26,28,30,31,33,34,41,44,45,47,48] and in 13 studies, taking medication (different classes of drugs in each experiment) were exclusion criteria [21,24,25,26,30,34,41,44,45,47,48,52]. Up to 11 studies did not define exclusion criteria [22,23,27,29,32,35,37,39,49,51]. 

The most often applied instrument to measure stress severity was the Perceived Stress Scale (PSS, n = 11) [21,22,23,24,25,26,29,32,44,45,52,54]. The few studies assessed stress using the Coddington Life Events Scale for Children (CLES-C, n = 3) [34,35,37], Strengths and Difficulties Questionnaire (n = 3) [34,35,37], Life Events Checklist (LEC, n = 2) [30,43], and Total Objective Hardship Score (Storm32, n = 2) [39,40]. Other questionnaires were not duplicated. The examined factors and the tools are presented in Table S1.

The majority of studies analyzed the connection between stress and body composition (n = 22) [21,22,24,25,26,27,28,29,33,34,36,38,41,44,45,46,47,48,49,50,51,53], body fat alone (n = 4) [23,42,43,52] or together with body composition analysis (n = 3) [28,33,46]. BIA was the most-often-used method (n = 11) for the assessment [27,29,33,36,41,44,45,47,48,51,53]. Several researchers performed body composition by DXA (n = 6) [22,25,26,33,46,50], plethysmography (n = 5) [21,24,28,35,38], and computed tomography (CT, n = 1) [49]. To determine body fat, magnetic resonance imaging (MRI, n = 3) [28,46,52], measurement of skinfold thickness (n = 3) [33,42,43], and the tape test (n = 1) were carried out [23].

For biochemical parameters, the most often examined was found to be cortisol (n = 9) [21,25,28,34,35,37,41,43,52]. However, researchers examined the concentration in different biological samples (plasma (n = 1) [41], serum (n = 1) [21], saliva (n = 5) [25,28,35,37,52], and hair (n = 2) [34,43] and using various methods (chemiluminescence assay (CLIA, n = 2) [43,52], competitive fluorescence enzyme immunoassay (FEIA, n = 1) [41], liquid chromatography with tandem mass spectrometry (n = 1) [34], automated enzyme immunoassay (n = 1) [28], electrochemiluminescence immunoassay (n = 1)) [35]. In three studies, the method of sample analysis was not determined [21,25,37]. 

In five studies, blood glucose levels were determined in serum (n = 2) [25,31] and plasma (n = 1) [50]. Two studies did not assess blood fraction using analysis [26,48], and only one indicates the examination method (enzyme-linked immunosorbent assay (ELISA) [48]. Glycosylated hemoglobin (HbA1c) as an indicator of average glucose level over the past 2–3 months was examined in one study without describing the applied method [53].

Five studies analyzed blood insulin in serum (n = 3) [25,31,50], and two did not determine the used blood fraction [26,48]. Using methods were: CLIA (n = 2) [25,48], radioimmunoassay (RIA, n = 1) [31], and microparticle enzyme immunoassay (n = 1) [50]. One study did not include information on the method applied to assessment [26].

C-reactive protein (CRP) was measured in two studies (one using ELISA assay) [45,53]. The other tested inflammatory variables were only assessed in no more than one experiment included in the review. Examined markers included various inflammatory factors (plasma tumor necrosis factor-alpha: TNF-α, serum interleukin-1 receptor antagonist: IL-1Ra and fecal calprotectin) [30,34,45]. In some studies, peptides that regulate food intake levels were included in the analysis: plasma ghrelin, plasma leptin, plasma neuropeptide Y (NPY), and high-molecular-weight adiponectin [30,34,45]. Additionally, markers of lipid metabolism (serum HDL cholesterol, serum triglycerides (TG) (two studies), [25,53], serum LDL cholesterol and total cholesterol [53], metabolome including lipid metabolism pathways [45]) and markers of oxidative damage (serum 8-hydroxyguanosine (8-OxoG), serum 8-iso-prostaglandin F2α (IsoP) [26], and microbiota metabolites (short chain fatty acids, SCFAs, in stool)) were examined [34]. One study examined routinely examined blood factors, such as complete blood count (CBC), liver enzymes, blood urea nitrogen (BUN), creatinine, and uric acid [53].

### 3.1. Risk of Bias

An analysis of the ROB was limited by restricted information being provided in some of the papers (see: Supplementary Table S1 and S3). Finally, most of the studies (n = 19) included in the systematic review were at high risk of bias [21,22,23,25,26,27,28,33,34,35,38,39,40,44,45,48,50,52,53], and one had a very high risk of bias [24]. One study was a randomized clinical trial, and assessment according to NOS was impossible [41]. Only 12 studies had high quality [29,30,31,32,36,37,42,43,46,47,49,51].

### 3.2. The Relationship between Experienced Stress and Body Composition 

Of 11 studies that analyzed the relationship between stress and anthropometric measurement via BIA, 4 did not report any link between variables [41,44,45,48]. In contrast, four studies found a positive association between body fat percentage (PBF) and stress levels [29,36,47,53]. Some studies have shown that stress interplays with other factors (such as physical activity, gender, and diet) to explain changes in PBF [27,33,36,53]. Surprisingly, in one study, higher stress was associated with lower body fat content [51].

According to plethysmography data, no correlations between stress and body composition were found in four studies [21,24,28,38]. However, in a study conducted by Michales et al. (2015), negative emotions were related to adiposity [35]. According to an analysis by the plethysmography method, lifestyle factors could be found to moderate the longitudinal stress-adiposity relation [28,35].

According to DXA analysis, stress was not directly related to body composition in three studies [22,25,33]. However, in one study, it was observed that more stressed girls had higher PBF [50], and in another, maternal distress (MS) during pregnancy was associated with higher childhood fat mass [46]. DXA also revealed that stress plays a crucial role as a moderator of changes in anthropometric measurements. For instance, the consumption of highly palatable food was significantly associated with truncal fat in stressed women only [26].

MRI studies confirmed the positive relationship between the severity of stress and body fat [28,46]. Interestingly, one study reported an inverse relationship between stress and subcutaneous and visceral fat in women and a positive between psychological tension and subcutaneous fat in men [52]. In one study, stress was related to greater skinfold thickness [42].

### 3.3. The Relationship between Experienced Stress and Biological Markers

In total, 15 studies analyzed metabolism-related substances from various body fluids. However, there needed to be more consensus on methodology and examined markers. Cortisol in plasma, saliva, and hair was not related to stress experienced stress according to five studies [25,34,41,43,52] and was unexpectedly negatively associated with stress in one study [21]. Cortisol has been proposed as a factor mediating body composition changes in populations vulnerable to stress [28,35].

For factors engaged in carbohydrate metabolism, no effect of stress on glucose [25,48,50], insulin [25,48], or the Homeostasis Model Assessment of Insulin Resistance (HOMA-IR) [47,50] was reported in three papers. In one study, stress correlated to HOMA-IR. However, the relationship was found in the group of Afro-American immigrants but not among individuals from this ethnic group living born in a non-native country [31]. The results of one study suggest that stress could mediate abnormal insulin sensitivity caused by improper diet [26]. Yamamoto et al. revealed stress was related to higher HbA1c among men [53].

Few researchers examined the relationship between stress and lipid metabolism. The papers showed that stress was unrelated to total cholesterol, HDL, LDL fractions and triglyceride levels [25,53]. However, one study found that higher triglyceride levels characterized women with higher distress. In more depth, metabolomic assays found an inverse relationship between stress and lysophosphatidylcholine.

Three papers did not support the relationship between inflammatory markers and distress [34,45,53]. However, in another study, higher plasma TNF-α levels were associated with more significant stress [30] and bacterial metabolites [34]. In women with chronic stress, basal 8-OxoG (a marker of oxidative stress) was significantly higher than those of low-stressed women. Moreover, only women who experienced higher severity of stress were vulnerable to DNA and lipid peroxidation (serum levels of 8-hydroxyguanosine (8-OxoG) and 8-iso-prostaglandin F2α level (IsoP)) [26]. 

High inconsistency in examined markers was found in the papers that assess the relationship between exposure to chronic stressors and peptides regulating food intake [26,45,48]. Plasma basal leptin, active ghrelin levels, and high-molecular-weight adiponectin were independent of the intensity of experienced stressors [45,48], whereas while experiencing chronic stress, higher levels of NPY were shown [26]. Routinely examined blood parameters (CBC, liver enzymes, BUC, creatinine, and uric acid) did not correspond to distress [53]. 

## 4. Discussion

This systematic review examined the effect of psychological stress (distress) on metabolic-related factors and anthropometric measures in healthy individuals. To the best of our knowledge, this is the first study to summarize the results of data from human studies. Despite the many papers included in this review, the interpretation of accumulated evidence is hampered. The main challenges are the use of different methodological approaches by researchers and the high diversity of the examined populations. 

In total, 13 of 26 studies (50%) did not report any relationship between stress and body composition or body fat [21,22,24,25,28,33,36,38,41,42,44,45,48], while 7 studies found a significant positive association between variables [28,34,35,45,46,49]. It should be noted that in only two studies was higher stress associated with decreased body fat [51,52]. Ambiguous results obtained by researchers might be the results of different methods to examine anthropometric data. Despite most of the researchers using BIA devices (11 papers), the equipment was from other companies. Many publications omitted information about measurement conditions (time of analysis, patient preparation). The examinators did not consider the menstrual cycle in women as a factor that could affect intrapersonal variability of body composition parameters. An analysis performed during the same menstrual cycle phase would increase the reliability of the results [55]. 

No consensus exists on factors reinforcing fat accumulation in stress-related mechanisms. However, certain studies indicate the effect of gender on the stress–body composition relation with greater vulnerability of women [50,52]. Some researchers provided that stress is a factor interplay with lifestyle to explain changes in the accumulation of body fat [26,27,28,33,35]. These results are supported by model studies that show the negative impact of unhealthy eating habits on body composition during chronic stress exposure [56,57]. 

Despite quite a large number of studies included in our analysis, most of them have a high risk of bias. Analyzed data from studies with high quality, only two out of eight found no relationship between stress and body fat [43,49]. Five highlighted the positive association [29,36,42,46,47], while one highlighted the negative relationship [51]. Overall, there is no consensus on how psychological stress affects anthropometric measurements and which environmental and individual factors affect body response.

We identified 16 studies investigating the link between stress and metabolism-related biological markers. The researchers were inconsequent on the substances tested as same as the analysis method. Most papers concerned cortisol, a well-known glucocorticoid hormone, often named the stress hormone. Prolonged exposure to stressors affects the adrenocorticotrophic hormonal (ACTH) system, triggering adrenal glands to produce the end-product of the HPA axis: cortisol. Cortisol is the most prominent and clinically relevant biomarker of stress response examination and monitoring [58]. Many factors act on cortisol levels in biological fluids. Increased hormone levels are connected to metabolic factors (obesity, dyslipidemia, hypertension, diabetes) [59]. 

Surprisingly, one study found a negative relationship between the compound and stress [21], while the vast majority (67%) report no effect (indirect or direct) between the variables [25,34,37,41,43,52]. The studies used various analytical techniques. Only one study assessment of cortisol response was by mass spectrometry, which currently is the preferred method to assay for obtained reproducible results [34,60]. Some researchers suggest that ELISA (used in one study) is the most promising, reliable, laboratory-based method [28,61]. Blood cortisol levels fluctuate hour-to-hour, with higher concentrations in the morning and lower in the evening [62]. Not all included studies reported the time of assessment. 

Long-term systemic cortisol exposure is hard to establish due to its protein-binding capacity and circadian fluctuations. According to Verspeek et al., urinary cortisol reflects the longer-lasting effect of a stressor exposition, while salivary levels represent circulating levels with a short time lag of several minutes [62]. None of the included studies examined levels of the hormone in the urinary. 

Some studies suggest that the most promising technique for assessing long-lasting stress exposure could be hair cortisol levels [63]. While the papers in this review did not fully support this connection, it presents an optimistic outlook for the future of stress research. Notably, researchers found a negative impact of post-traumatic stress disorder on cortisol in hair and no effect of perceived stress, depressiveness or social support [63]. In the later phases of distress, HPA axis response and secretion of glucocorticosteroids may be dampened [58]. Additionally, data from papers with a low risk of bias (only two studies) did not report the relationship between salivary or hair cortisol and exposure to chronic stress [37,42]. 

The positive link of cortisol with stress-related anthropometric (BMI, body fat) measurements supports the interaction and interplay between perceived stress, cortisol fluctuations and anthropometric measurements [64]. Therefore, those mentioned above should be assessed simultaneously [28,35]. 

Insulin has inhibitory activity on the HPA axis, and its higher levels may be compensatory mechanisms to inhibit “stress-axis” activity [65]. Acute psychological stress promotes impaired insulin secretion by gradually deteriorating pancreatic β-cell function [66]. In the acute response, adrenaline release leads to higher glucose levels in blood circulation from hepatic glucose stores. Additionally, altered glucagon and insulin secretion from pancreatic cells affect glucose levels [66]. Most studies included in the review found no relationship between distress and carbohydrate metabolism. However, one study revealed a correlation between stress and HOMA-IR. This relationship was particularly interesting as it was found in the group of Afro-American immigrants but not among individuals from this ethnic group living born in a non-native country [31]. These findings suggest that cultural and environmental factors may influence the impact of stress on carbohydrate metabolism. Furthermore, the results of one study indicate that stress could mediate abnormal insulin sensitivity caused by improper diet [26]. Stress is suspected to impair glucose metabolism and affect at least six molecular pathways [66]. Nevertheless, molecular changes do not always appear as glucose intolerance or can readily be seen in the other peripheral blood markers. This partially explains the relationship between experienced stress and standard laboratory parameters [53]. 

Another proposed disruption caused by long-term stress exposure is the changes in lipid metabolism. A key role in lipid metabolism is the liver. The interplay between chronic repeated stress and high-fat diet-induced non-alcoholic fatty liver disease (NAFLD) progression was noted. The studies found glucocorticosteroids (also related to stress, as mentioned above) alter hepatic lipid metabolism and worsen dyslipidemia induced by an improper diet [67]. Although animal experiments confirm chronic stress increases serum levels of total cholesterol, triglycerides, low-density lipoprotein cholesterol, and very-low-density lipoprotein cholesterol, this issue in humans needs further attention [68]. 

Distress-induced hyperlipidemia was accompanied by increased oxidative stress. Decreased hepatic antioxidant enzyme activities and increased in lipid peroxidation of the kidney, liver and heart were observed [69]. Included in our analysis study, Aschbacher et al. found synergistic effects of psychological stress and diet on oxidative stress parameters. Indeed, basal levels of 8-hydroxyguanosine were higher in the stressed women group than in women with low stress levels. Moreover, the negative impact of highly palatable foods appeared only in case of exposure to stressors [26]. 

From studies examining the pro/anti-inflammatory state included in this systematic review, only one study, with a low risk of bias (opposite to three others with a high risk of bias), found a positive link between these factors [30]. TNF-α was related to higher stress. The examination was performed on children with diabetes mellitus (also associated with inflammation) risk. However, other studies in individuals with higher inflammation susceptibility (due to high BMI index) showed contrary results [45]. It should also be noted that none of the assessed factors in the included studies were duplicated in two or more studies. Glucocorticosteroids, released in response to stress, may lead to the suppression of immune responses. However, hormones of the HPA axis also have inflammatory potential via activation of the innate immune system in response to harmful signals from the environment [49]. Studies show acute stressors upregulate immune responses, while chronic stressors inhibit them. Nevertheless, excessive stress affects overstimulation of the immune system, leading to disruption of pro-/anti-inflammatory homeostasis [49]. Indeed, the intensity and type of stressors might cause inconsistencies in the results and difficulty interpreting them.

The overload of stressors leads to changes in food intake and affects unhealthy dietary patterns. These behaviors might be a result or reason induced by disruptions in hormones engaged in food intake. Notwithstanding, the studies included in the systematic review do not support this hypothesis. Only one examination found a positive relationship between psychological stress and higher NPY levels [26]. NPY, also known as a PYY4, influences many physiological processes, including stress response, food intake, and circadian rhythms. There has been a suggestion that neurohormones protects from the negative impact of stress, such as disrupted behavior, anxiety and depression [70]. In cognitive behavioral therapy (CBT), dropping out of NPY levels is accompanied by a reduction in stress among women with fibromyalgia syndrome [71]. 

Despite the number of studies included in this systematic review, some limitations of our analysis should be mentioned. The main issue is that the studies that were included have observational (cross-sectional or cohort) designs. Only well-designed and well-conducted clinical studies may determine the direct impact of stress on anthropometric and metabolism-related markers with exclusion risk of reverse causation. It should be emphasized that there was no unified assessment of stress exposure, so the examined group could vary by intensity and type of stressors. The included papers reported other methodological tools to measure biochemical and anthropometric data. Different populations were included for assessment; not all researchers determined the inclusion and exclusion criteria of studies. The stress responses vary across populations or contextual factors (e.g., socioeconomic status, gender, cultural differences). Including these factors in further studies enables a more detailed understanding of the mechanisms underlying the relationship between psychological stress and chronic diseases. The investigators should especially concentrate on groups with high vulnerability to various stressors and try to use a similar methodology.

## 5. Conclusions

The vast majority of assessed studies do not support the effect of chronic distress on anthropometric measurements and biological markers levels. However, upon analyzing data from the most-well-designed study, most found that stress increases body fat content. More compliance with the assessed metabolism-related biomarkers regarding the method and type of assessed fluid needs to be made. Further, researchers should pay attention to applying similar assessment methods to stress’s intricate and complex effects on the human body. The synergistic effects of unhealthy lifestyle patterns and psychological stress should also be considered. Only in-depth evaluation, assessing biological factors on various levels (i.e., gene expression and regulation, protein concentration), or an untargeted analysis is capable of the relationship between chronic psychological stress and health-related outcomes. v

## Figures and Tables

**Table 1:** The general characteristics of the included studies.

Ref.	Authors, Date	Study Desing	Country	The Aim	ROB
[41]	Kyrou et al., 2017	Randomized double-blinded placebo-trial (RCT)	Greece	Explore the effects of a commercially available hops dry extract on self-reported depression, anxiety and stress levels.	N/A
[42]	Gerber et al., 2017	Cross-sectional study	Switzerland	Test whether physical activity and fitness moderate the relationships between different psychosocial stress indices and obesity-related measures.	7
[47]	Patel et al., 2019	Cross-sectional study	India	Assess dietary intakes, stress levels and PBF, study the interrelationships of PBF with stress, dietary fat, and carbohydrate.	7
[21]	Joseph et al., 2019	Natural history study (case–control)	United States	Examine the interrelationships among stress, eating behavior, and adiposity.	6
[22]	Hootman et al., 2018	Longitudinal prospective cohort study	United States	Evaluate sex differences in stress, emotional eating, tendency to overeat, and restrained eating behavior, and determine whether the psycho-behavioral constructs assessed immediately prior to starting college are associated with anthropometry and adiposity at the start of college, and with first-semester weight gain.	6
[48]	Macedo & Diez-Garzia, 2014	Observational, transversal study	Brazil	Identify sweet craving (SC) in women with stress, investigate how stress and SC affect the basal leptin and active ghrelin levels, assess the metabolic abnormalities that could influence ghrelin levels and that could be present in stress, and evaluate the body composition and fat distribution related with both stress and SC.	4
[23]	Jayne et al., 2020	Cross-sectional study	United States	Examined gender as a moderator and emotional eating as a mediator of associations between perceived stress, BMI, and failing body composition standards.	6
[24]	Wilson et al., 2019	Observational study	United States	Examine whether validated measures of anxiety and life stress relate to RMR in a sample of male and female adults.	3
[25]	Strait et al., 2018	Cross-sectional, pilot study	United States	Examines the degree to which insulin resistance, dyslipidemia and ectopic fat deposition correlate with chronic psychological stress and salivary cortisol measures of HPA axis activation.	4
[46]	Vehmeijer et al., 2019	Population-based prospective cohort study	Netherlands	Assess the association between maternal psychological distress and childhood organ fat measures.	7
[38]	Hruska et al., 2020	Pilot randomized control trial	Canada	Investigate the associations between three measures of stress (general stress, parenting distress, and household chaos), and adiposity.	6
[26]	Aschbacher et al., 2014	Cross-sectional and prospective study	United States	Assess the synergistic effects of psychological stress and diet on oxidative stress, insulin resistance and adiposity.	5
[50]	Olive et al., 2017	Longitudinal cross-sectional study	Australia	Investigate the links between psychosocial stress and depressive symptoms with insulin resistance.	6
[27]	Shin et al., 2016	Secondary analysis of data	United States	Examine the association of psychological stressors with cardiometabolic risk factors (blood pressure, BMI, and body fat) and determine the moderating effect of coping between racial discrimination and those risk factors.	6
[44]	Fāohr et al., 2016	Longitudinal study	Finland	Investigate the effects of physical activity and objective stress and recovery on subjective stress over a 9-month study period.	6
[34]	Michels et al., 2017	Cross-sectional study	Belgium	Verify the association of chronic stress with gut health.	4
[28]	Donoho et al., 2011	Cross-sectional study	United States	Examine the independent and interactive effects of stress and HPA axis activity in predicting visceral adipose tissue and subcutaneous abdominal adipose tissue.	5
[35]	Michels et al., 2014	Cross-sectional study	Belgium	Examine the bidirectional longitudinal association between stress and adiposity and test moderation in the stress-adiposity relation by both life-style parameters and cortisol levels.	6
[29]	Isasi et al., 2015	Population-based cohort study	United States	Examine ongoing chronic stress in important life domains and perceived stress during the past 30 days in relation to obesity and dietary intake.	7
[36]	De Vriendt et al., 2012	Cross-sectional study	Six cities from European countries: Belgium, Sweden, Austria, Hungary, Greece, Spain	Describe the current perception of stress in a sample of European adolescents and to examine the hypothesis that perceived stress is associated with higher adiposity levels in adolescents.	7
[37]	Vanaelst et al., 2014	Cross-sectional study	Belgium	Examine the association between psychosocial stress and body composition.	8
[30]	Dixon et al., 2009	Cross-sectional study	United States	Determine potential predictors of TNF-α levels within a population of children who remained at increased risk for both insulin resistance syndrome and diabetes mellitus.	7
[51]	Wu et al., 2018	Prospective cohort study	Mexico	Investigated whether exposure to prenatal maternal stress, summarized as an index for multiple stressors, was associated with childhood adiposity measures and whether this association was different among males and females and may be mediated or modified by methylation in inflammation-related genes in umbilical cord blood.	7
[49]	Kim et al., 2017	Data from population-based cohort study	Korea	Verify the unidentified connection between RGS6 gene and human obesity with psychosocial stress.	7
[31]	Tull et al., 2003	Population-based study	United States	Determine whether acculturation and psychosocial stress exert differential effects on body fat distribution and insulin resistance.	7
[32]	Suglia et al., 2017	Data from the National Longitudinal Study/cross-sectional study	United States	Explore the association between perceived stress and adiposity (BMI and WC).	7
[39]	Liu et al., 2016	Prospective longitudinal study	Canada	Determine how associations between prenatal maternal stress (PNMS) and adiposity change with age.	6
[43]	Gerber et al., 2017	Large-scale cross-sectional study	Switzerland	Expand upon previous research by shedding new light on the associations between hair cortisol, social and demographic background, stress, and a series of health-related outcomes.	7
[53]	Yamamoto et al., 2007	Cross-sectional study	Japanese	Examine the relationship between the stress and biomedical parameters, such as anthropometric measurements, blood pressure, liver function tests, serum lipids concentrations, plasma glucose concentrations and the value for glycosylated hemoglobin, among apparently healthy subjects.	5
[45]	Noerman et al., 2020	Lifestyle intervention study	Finland	Examine whether the improvement of psychological well-being is associated with specific profiles of circulating metabolites that would indicate both subjective and objective measures of psychological well-being.	6
[52]	Jones et al., 2016	Cross-sectional study	United Kingdom	Study the relationship between the acute mental stress response and chronic stress exposure.	6
[40]	Cao-Lei et al., 2015	Prospective longitudinal study	Canada	Test the effects of objective and subjective PNMS and other risk factors on BMI and central adiposity in 13½-year-old adolescents and determine which DNA methylation could mediate the impact of PNMS on these outcomes	6
[33]	Monthé-Drèze et al., 2023	Prospective cohort study	United States	Investigate the longitudinal associations of the Dietary Inflammatory Index (DII) in pregnancy with growth and adiposity from childhood to early adolescence and examine the extent to which prenatal psychosocial stress may modify these associations.	6

ROB—risk of bias (points); N/A—not applicable; PBF—percentage body fat; BMI—body mass index; RMR—resting metabolic rate; HPA axis—hypothalamic-pituitary-adrenal axis; TNF-α—tumor necrosis factor alpha; WC—waist circumference.

## Data Availability

The data presented in this study are available by contacting the corresponding author.

## Supplementary Materials

The following supporting information can be downloaded at: https://www.mdpi.com/article/10.3390/medicina60081253/s1, Table S1: The general characteristics of the included studies; Table S2: The characteristics of the studies participants; Table S3: Risk of bias assessment.
