# Supplementary material for: The Relationship between Psychological Stress and Anthropometric, Biological Outcomes: A Systematic Review
Source: Medicina (Kaunas). 2024 Aug 1;60(8):1253. doi: 10.3390/medicina60081253 (PMC11356149; doi:10.3390/medicina60081253)
Supplement: Supplementary file 1 [file medicina-60-01253-s001.zip › medicina-3056462-supplementary.pdf]

Table S1. Characteristics of the using tools, the evaluating outcomes and main findings for the studies included to the systematic review.

Ref.	Exposure	Outcomes		Results	Conclusion
	Method for stress measure	Factor	Tool/method for measure		
[41]	Depression Anxiety Stress Scale-21 (DASS-21)	Body composition<br>Plasma cortisol levels	Bioelectrical impedance analysis (BIA) (BIA-ACC, Biotekna)<br>Competitive fluorescence enzyme immunoassay (FEIA)	1) No correlations were identified between any of the three DASS-21 subscale scores and baseline or post-treatment study variables.	Not related to aim of the study.
[42]	Questionnaire for Children and Adolescents for the Assessment of Health-Related Quality of Life—Revised Version (KINDL-R questionnaire)	Body fat<br>Physical activity<br>Fitness (endurance performance)	Skinfold thickness<br>Defined by parents as time spent in vigorous activities and sport, except physical education lessons [$min \cdot d^{-1}$]<br>20-m shuttle run test	1) School stress was positively correlated with: body fat (R=0.20; p<0.001), waist circumference (R=0.17; p<0.01);<br>2) School stress interacted both with fitness and physical activity to explain variance of body fat, and waist circumference.	High fitness is associated with less unfavorable body composition among children with elevated school stress.
[47]	International Stress Management Association questionnaire	Body composition<br>Diet	BIA (SC240 MA, Tanita)<br>24-h recall: 2 weeksday and Sunday	1)Percent of body fat (PBF) had a significant positive association with: stress levels (p = 0.02) and carbohydrate intake (p =0.008);<br>2)Women with moderate stress had 1.3 times, and women with high stress had 1.6 times higher PBF compared to women with less stress.	Women with higher stress and carbohydrate intake are more likely to accumulate higher body fat as compared to women with less stress and low carbohydrate intake.

[21]	Perceived Stress Scale with 14 items (PSS-14)	Body composition<br>Serum cortisol levels<br>Eating behavior	Air displacement plethysmography (BOD POD Body Composition System)<br>N/A<br>Three-Factor Eating Questionnaire (TFEQ)	1)No relationship between body fat and cortisol or perceived stress (the relationship between adiposity and eating behavior was only found).<br>2) Stress and cortisol levels were negatively associated (R=-0.21; p=0.32)	The need for stress management in patient support, particularly in nutritional and weight loss programs.
[22]	PSS-10	Body composition<br>Diet<br>Eating competence<br>Eating behavior<br>Physical activity	Dual energy X-ray absorptiometry (DXA) (QDR4500 fan beam densitometer, Hologic)<br>Diet History Questionnaire, (DHQ-II)<br>Satter Eating Competence Inventor (ecSI)<br>TFEQ<br>Global Physical Activity Questionnaire (GPAQ)	1)Stress was inversely associated with waist circumference (WC) (β: −0.32; p=0.029) in females;<br>2)Stress at study baseline was associated with WC changes in weight:<br>in males: 6.7 higher baseline stress score was associated with a 1.1 cm greater change in WC over the first semester compared to their counterparts with lower stress.	Males with higher perceived stress at college entrance subsequently gained significantly more weight in the first semester, but no such relation was evident in females.
[48]	Stress Symptoms Inventory Lipp (ISSL)	Body composition<br>Active ghrelin plasma level<br>Basal leptin plasma level<br>Blood glucose level<br>Blood insulin level<br>Sweet craving characterization	BIA (Biodynamics 450)<br>Colorimetric assay<br>Enzyme-linked immunosorbent assay (ELISA)<br>Chemiluminescence assay (CLIA)<br>Questionnaire for Assessment of Sweet Substance Dependence and Abuse	1)WC was significantly larger among stressed women (p=0.02);<br>2) Grouping the women according to the presence of stress revealed no statistical differences in terms of body fat;<br>3) No differences in plasma basal leptin, active ghrelin, insulin, and fasting glucose levels as well as HOMA index in the groups with	Stressed women are more prone to sweet craving, and this condition is associated with increased basal leptin levels, larger hip circumference, and altered body composition.

				high or low stress.	
[23]	PSS-10	Body fat<br>Emotional eating	Tape test and body composition failure<br>Intuitive Eating Scale-2 (IES-2)	1)Perceived stress may affect body composition failure (OR=1.04, 95% CI:1.01-1.08, β=0.03); p<0.05;<br>2)Emotional eating is a mediator of relationship between stress and body fat (OR=1.02, 95%CI: 1.01-1.03 β:0.03); p<0.05.	Stress should be considered a harmful influence on force readiness, and identified emotional eating as a behavior that may mediate the relationship between stress and body weight.
[24]	PSS-14	Body composition<br>Resting metabolic rate (RMR)<br>Anxiety	Air displacement plethysmography (BOD POD Body Composition System)<br>The ventilated canopy method (TrueOne 2400, ParvoMedics)<br>The State-Trait Inventory for Cognitive and Somatic Anxiety (STICSA)<br>Anxiety Sensitivity Index (ASI)-3	1) No relationship between RMR, body composition and perceived stress.	Anxiety, and life stress do not have substantial associations with RMR in individuals without anxiety disorders.
[25]	PSS-10<br>Positive and Negative Affect Schedule for Children (PANAS-C)	Body composition<br>Serum glucose level<br>Serum insulin level<br>Serum HDL cholesterol level<br>Serum triglycerides level<br>Serum LH level<br>Salivary cortisol levels	DXA (XR-800 whole body bone densitometer, Norland)<br>N/A<br>CLIA<br>N/A	1)No significant correlations of chronic self-reported stress and metabolic syndrome risk markers;<br>2) No significant correlations of chronic self-reported stress and salivary cortisol.	Relationships between psychological stress, activation of the HPA axis, deposition of ectopic fat and risk for metabolic syndrome remain unresolved.

		Bullying	Social Experience Questionnaire (SEQ)		
[46]	Brief Symptom Inventory (BSI)<br>Global Severity Index (GSI)	Body composition<br>Body fat deposition	DXA (iDXA, GE140 Lunar, 2008, Madison, WI)<br>3.0 Tesla Magnetic resonance imaging (MRI) (Discovery MR 750w; GE Healthcare)	1)Maternal overall psychological distress (MS) during pregnancy were associated with higher childhood fat mass (FM) index (measures independent of height) ($p<0.05$);<br>2)After adjustment for potential confounders, MS was associated with higher childhood FM index (difference 0.14 (95%CI:0.04–0.24));<br>3)MS were associated with higher childhood subcutaneous fat index and liver fat fraction ($p<0.05$), but this relationship unremained significant after adjustment for potential confounders;<br>4) Associations of MS with childhood pericardial fat index was not observed.	Maternal psychological distress during pregnancy is associated with higher general fat measures and anxiety with higher general and organ fat measures in children.
[38]	Parenting Stress Index (PSI)	Body composition<br>Home environment chaos	Air displacement plethysmography (BOD POD Body Composition System)<br>Confusion, Hubbub and Order Scale (CHAOS)	1) Parenting distress was positively associated with waist circumference (β=0.39; 95%CI: 0.04-0.7) and WHR (β=0.003; 95%CI: 0.001-0.005);<br>2)No association of body fat and parenting distress was found.	Stress is associated with adiposity in parents of young children. Stress, parenting distress, and stress in the home environment may be important determinants of parental

					weight outcomes.
[26]	PSS-10	Body composition<br>Plasma Neuropeptide Y (NPY) level<br>Blood glucose level<br>Blood insulin<br>Serum 8-hydroxyguanosine (8-OxoG) level<br>Serum 8-iso-prostaglandin F2α level (IsoP)<br>Diet	DXA (Lunar Prodigy densitometer)<br>ELISA<br>N/A<br>Liquid chromatography with tandem mass spectrometry<br>29-item version of the Block Food Frequency Questionnaire (FFQ)	1) Basal 8-OxoG was significantly higher among women with chronic stress (CS) compared low-stressed (p<0.01);<br>2) Chronic stress (CS) group had significantly higher levels of basal NPY (p<0.01);<br>3) Only in CS woman consumption of highly palatable food was significantly associated with WC, truncal fat, insulin sensitivity, 8-OxoG, and IsoP at baseline, 1 year after and the longitudinal change terms (p<0.05).	CS is associated with enhanced vulnerability to diet-related metabolic risk (abdominal adiposity, insulin resistance, and oxidative stress). Stress-induced peripheral NPY may play a mechanistic role.
[50]	Children's Stress Questionnaire (CSQ)	Body composition<br>Serum insulin levels<br>Plasma glucose levels<br>Severity of depression symptoms<br>Physical activity	DXA (Hologic Discovery QDR Series; Hologic)<br>Microparticle enzyme immunoassay<br>N/A<br>Children's Depression Inventory (CDI)<br>New Lifestyle pedometers (Lee's Summit, MO)	1)No evidence for a longitudinal effect of stress on HOMA-IR or percentage of body fat for boys and girls;<br>2)Associations between perceived stress and HOMA-IR were not significant at the cross- sectional level for either boys or girls;<br>3)Girls with greater stress had higher PBF (β=0.033; p=0.020).	Not related to aim of the study.
[27]	Weekly Stress Inventory (WSI)	Body composition<br>Resting systolic blood pressure (SBP) and diastolic blood pressure (DBP)	BIA (Tanita)<br>N/A	1)No significant associations were found between psychological stressors (i.e., discrimination, stress) and PBF;	Perceived stress was associated with percentage of body fat in African American and

		Experience of racial discrimination	Modified Experiences of Discrimination questionnaire (EOD)	2)Racial discrimination (β =-0.529; p=0.032) and stress (β =0.175; p=0.033) were significant contributors to PBF in addition to age (β=0.340; p<0.001) and this model explained 14.1% of the variance in PBF.	Hispanic/Latina women.
[44]	PSS-14	Body composition<br>Stress index and recovery index<br>Physical activity	BIA (InBody720/Tanita BC-418)<br>Heart Rate Variability (HRV; over 1-3 days)<br>Validated questionnaire (no information which) and calculation of metabolic equivalents (METs) (hour/day)	1)The initial levels of the METindex (standardized β=0.266; p<0.001), stress index (standardized β =0.259; p=0.001) and recovery index (standardized β = 0.250; p<0.01) were significantly associated with the change in PSS;<br>2)Higher initial level of the stress index predicted a weaker decline in PSS, higher initial levels of the recovery and MET indices predicted larger decline in PSS during the study period.<br>3) PBF was no associated with changes in PSS.	Not related to aim of the study
[34]	Coddington Life Events Scale for Children (CLES-C)<br>Strengths and Difficulties Questionnaire (parents)	HRV<br>Hair cortisol analysis<br>Fecal short chain fatty acids (FCAs)<br>Fecal calprotectin	N/A<br>Liquid chromatography with tandem mass spectrometry<br>Gas chromatography<br>Fluoro Enzym immunoassay	1) From the stress measures, only emotional problems were related to butyrate (β=0.272; p=0.004), isobutyrate (β=0.209; p=0.026) valerate (β=0.238; p=0.011) and isovalerate (β=0.215; p=0.022) SCFAs.	Emotional problems were associated with higher butyrate, valerate, isovalerate and isobutyrate. Stress was not associated with calprotectin.

[28]	Stressful life events	Body composition<br>Body fat deposition<br>Salivary cortisol levels	Air displacement plethysmography (BOD POD Body Composition System)<br>MRI (Siemens Magnetom 1.5T Symphony Maestro Class Syngo 2004A)<br>Automated enzyme immunoassay	1)School-related life events was consistently related to VAT (MRI data) (β=0.39; p=0.01);<br><br>2) The interaction of school stress and cortisol accounted for:<br>- over 9% of the VAT variance after accounting for PBF, Tanner stage and the independent effects of school-related life events and cortisol (ΔR^2=0.09, F(1, 17)=4.64,; p=0.05, f2=0.26);<br>- over 14% of the SAT variance after accounting for PBF, Tanner stage and the independent effects of school-related life events and cortisol (ΔR^2=0.14, F(1, 17)=7.86; p=0.01, f2 = 0.49)<br>-over 13% of the PBF variance after accounting for abdominal fat, Tanner stage, and the independent effects of school-related life events and cortisol (ΔR^2=0.13, F(1, 17) = 5.62; p=0.03, f2 = 0.32).<br><br>3) For girls with high CAR, school-related life events was associated with increased VAT and SAT.	Regression analysis indicated an interaction of school-related life events and cortisol on VAT and SAT, with greater numbers of school-related events being related to greater VAT and SAT for girls with high cortisol, but no association with VAT or SAT for girls with low cortisol. Similar to job stress in adults, school-related stress in children may contribute to central adiposity, especially for girls with high cortisol.
[35]	Summing up the scores of the three stress aspects per	Body composition	Air-displacement plethysmography (BOD POD Body Composition System)	1)PBF could influence the overall reported stress level over time (p<0.001) but reported stress not	The results of demonstrate the importance of moderation: stress is

	child:<br>CLES-C,<br>the negative emotions score and Strengths and Difficulties Questionnaire (parents)	Salivary cortisol levels<br><br>Diet<br><br>Eating behavior<br><br>Physical activity	After wake up (T0), 30 minutes after wake up (T30), 60 minutes after wake up (T60), and in the evening by competitive electrochemiluminescence immunoassay<br><br>Food Frequency Questionnaire validated for the European IDEFICS study<br><br>Dutch Eating Behavior Questionnaire<br><br>Accelerometers worn at the hip	influences adiposity longitudinally and only negative emotions were found to be related to consequent adiposity;<br><br>2)In case of high cortistol level there was a positive longitudinal association between reported stress and central adiposity (WHtR) but only the negative emotions provided significant results;<br><br>3) Life-style factors could moderate the longitudinal stress-adiposity relation: for percentage of fat moderate-to-vigorous activity (t=-2.122; p=0.034), sedentary time (t=-2.084; p=0.037) and WHtR (t=2.002; p=0.048);<br><br>4)PBF seemed to cause higher levels of stress in children with frequent external eating but not in those with low external eating (t=0.058; p=0.013).	associated with increased adiposity when children have high cortisol awakening patterns and/or an unhealthy life-style. Because life-style behavior can also be applied as a coping mechanism and because it moderates the stress-adiposity relation, multifactorial obesity prevention programs should be created by focusing concurrently on stress and life-style. Children and their parents should realize the effects of stress on their diet, and an activity-friendly environment should be created to minimize sedentary time.
[29]	PSS-10<br><br>Chronic stress burden	Body composition<br><br>Diet and overall diet quality<br><br>Severity of depression symptoms<br><br>Physical activity	BIA (TBF 300, Tanita<br><br>24-recall and the alternative healthy eating index (AHEI-2010)<br><br>Center for Epidemiological Studies Depression Scale, (CES-D10)<br><br>World Health Organization (WHO) Global Physical Activity Questionnaire (GPAQ)	1) WC: β=3.3 (1.0-5.5) and PBF: β=1.5 (0.4, 2.6) increased with the number of chronic stressors reported;<br><br>2)Perceived stress during the past month was not associated with WHR or BF.	There is an association between stress and obesity and adiposity measures, suggesting that stress management techniques may be useful in obesity prevention and treatment programs that target Hispanic/Latino popu-

					lations
[36]	Adolescent Stress Questionnaire (ASQ)	Body composition<br>Diet<br>Physical activity	BIA<br>24h recall<br>Diet quality index for adolescents (DQI-A)<br>Accelerometers	1)In the girls, stress ASQ summary score was significant positive predictors for body fat (BF) (β=0.02; p=0.003);<br>2) When additionally controlling for their diet quality, the DQI-A itself was no significant predictor, while it attenuated the significance of the stress (β=0.017; p=0.020);<br>3) When additionally controlling for physical activity, the significant positive associations remained (β=0.024; p=0.001) while the level of PA itself was a significant negative predictor (β=-5.596; p=0.005);<br>4)The boys' ASQ summary score was no significant positive predictor for their measures of adiposity but their physical activity were negative predictor of stress (β=-5.986; p=0.007).	Adolescent boys and girls showed similar profiles of stress. Only in girls, perceived stress was positively associated with measures of general and abdominal adiposity, supporting the hypothesis that during this early life phase of adolescence, stress might be involved in the etiology of obesity.
[37]	CLES-C	Salivary cortisol levels	Two consecutive weekdays at four time points: awakening (T0), 30 min after waking up (T30), 60 min after waking up (T60) and between 7 and	1) A positive association was shown for children WHtR with the negative events score for the past 6 months (β=0147; p=0.013);<br>2) The positive association between	Children's stress level, characterized by negative life events, happiness and peer problems, was associated with overall

	Basic Emotions		9 pm (Tev) by	stress and WHtR was strengthened by unhealthy behavior (high-caloric snack frequency, high screen time, low sleep duration).	and central adiposity. Lifestyle factors did not mediate this relationship, although they were shown to intervene as moderators. An unhealthy lifestyle may make children more prone to stress-induced changes in body composition or, vice versa, a healthy lifestyle may attenuate the effects of stress on adiposity.
	Strengths and Difficulties Questionnaire (parents)	Diet	Children's Eating Habits Questionnaire-Food Frequency Questionnaire (CEHQ-FFQ)		
[30]	Life Events Checklist (LEC)	Plasma tumor necrosis factor alpha (TNF-α) level	ELISA	1) Higher plasma TNF-α levels were associated with greater stress (R=0.26; p=0.01).;<br><br>2)Multiple regression analyses revealed that BMI (β=–0.26; p<0.01) and stress (β=0.21; p<0.05) each contributed a significant proportion of unique variance associated with TNF-α levels;<br><br>3)When multiple regression analyses were conducted by entering family history of DM (selection variable) simultaneously into the equation with BMI, stress, and sex, no main effect was determined;<br><br>4)When multiple regression anal-	BMI and stress as independent determinants of TNF-α (an inflammatory cytokine and adipocytokine) among Latino children.

				yses were conducted by entering sex (selection variable) simultaneously into the equation with BMI and stress, no main effect was determined.	
[51]	Composite stress index based on four stress scales:<br>The Exposure to Violence (ETV) questionnaire, The Crisis in Family Systems-Revised (CRISYS) survey, Edinburgh Postnatal Depression Scale (EPDS) and the Speilberger State-Trait Anxiety Inventory	Body composition<br>Cord blood DNA methylation of TNF α<br>Cord blood DNA methylation of IL6<br>Cord blood DNA methylation of IL8	BIA (InBody 370 or 230 Biospace)<br>The pyrosequencing assay	1) An increase of stress index (IQR=27.3) was associated with decreases in 3.5% in PBF (95% CI: −6.3, −0.5), and 1.2% in WC (95% CI: −2.4, −0.04);<br><br>2) This associations appeared stronger among girls than among boys. In particular, there were a significant 9.0% decrease in FM and a significant 6.4% decrease in PBF in girls, compared to the insignificant decreases of 2.6% and 1.4% in boys, respectively;<br><br>3)The negative associations between stress index and offspring adiposity measures appeared greater among children with birth weight lower than the median (3.1kg) than among children with birth weight above or equal to the median;<br><br>4)The stress index were generally not associated with methylation in TNF-α, IL6 or IL8. except that EPDS was associated with a significant increase in IL6 methylation (β=0.81, 95%CI (0.25, 1.37)).	A negative association between prenatal maternal stress and adiposity in children and a positive association between methylation in IL6 promoter in cord blood leukocyte DNA and adiposity in early childhood, which was independent of prenatal stress were documented.

[49]	Psychosocial Well-being Index short form (PWI-SF) questionnaire	Body composition<br>Comprehensive adiposity phenotypes<br><br>Single nucleotide polymorphism of RGS6 gene	Computed tomography<br>Visceral adipose tissue (VAT) value from and anthropometric data<br><br>DATA from GWAS study Affymetrix Genome-Wide Human SNP array 5.0	1)The SNP rs2239219 was nominally associated with WC by interacting with stress (p=0.029);<br><br>2)Three RGS6 SNPs and stress level (rs2681749 OR=0.57, 95%CI:0.33-0.97; p=0.04, rs847330 OR=0.37, 95%CI:0.15-0.91; p=0.03, and rs2239219 OR=2.21, 95%CI:1.24-3.92; p=0.07) showed nominal interaction effects with WC but no with PBF;<br><br>3) Variant (homozygote) rs2239219 of gene increase 14.61% abdominal obesity (based on WHR) in case of highly stressful conditions compared low or moderate. This SNP was significantly associated with the prevalence of abdominal obesity in high stress group (p<0.05), whereas significant association in low-moderate stress group was not observed (p>0.05).	SNP rs2239219 of RGS6 is closely linked to stress-induced abdominal obesity, emphasizing the importance of stress management on prevention of obesity in Korean adult.
[31]	Roger's Life Attitude Inventory (LAI) questionnaire	Serum glucose levels<br><br>Serum insulin levels<br>Diet<br><br>Physical activity	Ektachem 700 Analyzer (Eastman Kodak Company, Rochester)<br>Radioimmunoassay (RIA)<br><br>Non-validated questionnaires	1)Psychosocial stress was not significantly correlated to the WC but correlated with lnHOMA (only among African-Caribbean immigrants), also a independent of acculturation score;<br><br>2) In the multiple regression analysis model that included WC this relationship un-	Acculturation and psychosocial stress may have a differential effect on body fat distribution and insulin resistance among native-born and immigrant blacks living in the US Virgin Islands. Psy-

				remainded significant.	chosocial stress may be a significant risk factor for some individuals and should be considered when lifestyle modifications are being recommended.
[32]	PSS-4	Severity of depression symptoms<br><br>Physical activity	The 10-item Center for Epidemiologic Studies Depression Scale (CES-D)<br><br>Unvalidated questionnaire participants were asked how often they had engaged in a number of physical activities for any duration of time in the past 7 days	1)The association between perceived stress and WC was not statistically significant in the partially adjusted model (adjusting for sex, race/ethnicity, age, and education) or the fully adjusted model (adjusting for sex, race/ethnicity, age, physical activity, smoking, education, sleep, and depressive symptomatology), but was significant (β:−0.80, SE:0.19; p<0.0001) when the cross-product term for the interaction of perceived stress and sex was included.<br><br>2) As perceived stress increases, WC decrease for both men and women but a steeper decrease was noted among men compared to women;<br><br>3)For every unit increase in perceived stress among men, WC decreased 0.21 cm (SE = 0.10), when adjusting for race/ethnicity, age, and education and this association	Perceived stress was associated with lower levels of adiposity among men. Noted differences could be attributed to different behavioral and coping strategies in response to stress between men and women as well as biological mechanisms which should be explored further.

				was not statistically significant among women (β=0.10, SE = 0.05);<br><br>4)Further adjusting for sleep duration and depressive symptoms did not change the results for WC among men or women (men β:−0.21, SE: 0.10; women β: 0.001, SE: 0.12);<br><br>5) The association was no longer statistically significant in men when further adjusting for physical activity and smoking (β:−0.18, SE:0.10).	
[39]	Impact of Event Scale – Revised<br><br>Objective PNMS/ Total Objective Hardship Score (Storm32) (Mothers)	Cognitive appraisal	No validated scale	1)The correlations between body composition and maternal subjective distress tended to increase with age and was significant for WHtR ratio at ages 11½, (R=0.25; p=0.04) 13½ (R=0.32; p=0.01), and 15½ (R=0.29; p=0.04);<br><br>2)A significant three-way interaction between objective hardship, subjective distress and age of the child was detected (p=0.037) indicating the effect of subjective distress on the change in central adiposity with age depends on the mother's level of objective hardship during the ice storm;	Higher PNMS was associated with higher central adiposity in children and the effects of PNMS on anthropometric measurements tend to increase as the children grew older.

				3)Probing the interaction revealed that, for women who were exposed to a low objective hardship, the effect of subjective stress on central adiposity did not vary with age, and that effect was never significant;<br><br>4)For women with high levels of objective hardship, starting at an objective stress score of 12, the effect of subjective stress on central adiposity increased significantly as their children got older.	
[43]	LEC<br>The Survey of Middle School Stressors	Body fat<br>Blood pressure<br>Static retinal vessel analysis<br>Cardiorespiratory fitness/physical performance<br>Hair cortisol analysis<br>Health-related quality of life<br>Psychosomatic complaints	Skinfold thickness<br>Automated oscillograph (Oscillomate, CAS Medical Systems)<br>The Static Retinal Vessel Analyzer (SVA-T, Impedos Systems UG)<br>The 20 m shuttle run test<br>CLIA<br>Questionnaire for Children and Adolescents for the Assessment of Health-Related Quality of Life – Revised Version (KINDL-R questionnaire)<br>8 items from Health Behaviour in School-aged Children checklist (HBSC-SCL)	1) Experienced stress no affected on levels of hair cortisol;<br><br>2)The regression analysis of girls subgroup showed BMI was the only significant predictor of cortisol in girls (β=0.22; $p<0.001$, R^2=0.027), boys subgroup somatic complaints remained the only significant predictor variable (β=0.20; $p<0.05$, R^2=0.023);<br><br>3)In the whole sample, body weight was the only significant predictor of cortisol (β=0.16; $p<0.01$).	Despite small significant relationships between hair cortisol, BMI and somatic complaints, the findings challenge the view that cortisol can be used as a reliable biomarker of recent critical life events, daily hassles, health-related quality of life, or cardiovascular health in a sample of non-clinical young children.

[45]	PSS-14	Body composition	BIA analysis (In Body 720 or Tanita BC 418)	1)The inverse relationship between stress and (lyso)phosphatidylcholine (14:0/16:0), and (20:4) was found.	The findings allow us to generate hypothesis on specific metabolites and the potentially relevant metabolic processes which could be involved in the systemic response to stress relief. Findings provide supportive evidence for further investigations aiming to unveil the role of the identified phospholipids and another class of unidentified lipids in the molecular interplay between psychological well-being and metabolic health, including other related factors such as body composition and inflammation.
		Serum high molecular weight adiponectin levels<br>Serum high sensitivity C-reactive protein levels (hs-CRP)<br>Serum interleukin-1 receptor antagonist levels (IL-1Ra)	ELISA		
		Metabolic profiling of plasma samples	LC-MS analysis		
		Objective stress and relaxation	HRV		
		Vitamin A intake	48h recall		
		Alcohol consumption	Alcohol Use Disorders Identification Test-Consumption (AUDIT-C) questionnaire		
		Psychological flexibility	Acceptance and Action Questionnaire for Weight-related difficulties (AAQW)		
		Physical activity	Questionnaire covering leisure-time and commuting activity and presented at MET/hour		
		The sleep quality	7 days sleep diary		
		The sleep efficiency	An actigraphy measurement for one week		
[53]	Inventory to Measure Psychosocial Stress (IMPS)	Body composition	BIA (Omrom, HBF302)	1) The stress was linear associated with PBF (p=0.001) and HbA1c among men (p=0.018);<br><br>2) When the relationship between stress and PBF	The stress score was associated with obesity and unfavorable glycemic changes is in parallel with the model that psychosocial stress has a
		Glycosylated hemoglobin (HbA1c)<br>Total, HDL, LDL cholesterol level<br>Triglycerides level	N/A		

		Liver function test<br>CRP<br>Blood urea nitrogen (BUN)<br>Creatinine<br>Uric acid<br>Complete blood count (CBC)<br>Blood pressure		was controlled for alcohol consumption and smoking, the significant correlation attenuated (p=0.057), and was nullified when exercise entered into the analysis (p=0.144);<br><br>3) The stress among men remained correlated with the value for HbA1c even after controlling for age, BMI, alcohol consumption, smoking, and exercise (p=0.018);<br><br>4) There was a linear relationship between the stress score and triglyceride concentrations (p=0.001) among women, also controlling for age and BMI (p=0.009);<br><br>5) The correlation between stress and triglyceride concentration among women was nullified after controlling for age, BMI and alcohol consumption (p=0.132).	detrimental effect on humans by inducing obesity and insulin resistance.
[52]	PSS (no information about version)	Body fat deposition<br>Cardiac function<br>Salivary cortisol levels<br>Acute stress	1.5-T MRI (Avanto, Siemens)<br>CLIA<br>Montreal Imaging Stress Task (MIST) to induce stress and MRI measurement	1)Greater PSS scores in women were associated with lower percentage of subcutaneous (p=0.04) and visceral fat (p=0.01) but with higher subcutaneous fat in men (p=0.04). These sex differences were significant on interaction testing;	The gender demonstrate marked differences in cardiovascular, neuroendocrine and metabolic function when exposed to chronic stress. These

		Aortic flow	Accelerated ECG-gated spiral phase-contrast MRI sequence	2) Rest and peak post-stress cortisol were not associated with PSS in either sex but the difference between them (stress response) was smaller in women with greater PSS (p=0.01).	differences have important implications for stress-related disorders such as obesity, diabetes and cardiovascular disease.
		Blood pressure	N/A		
		Physical activity	Baecke short questionnaire		
[40]	Impact of Event Scale – Revised (IES-R): subjective (M)<br>Storm32 (Mothers)<br>Timing of exposure to the ice storm in utero (Children)<br>Life Experiences Survey (LES) (mothers)	DNA methylation levels in T-cells (mothers)	Infinium Human Methylation 450 BeadChip Array	1)Objective (R=0.326; p=0.012) and subjective (R= 0.307; p=0.018) PNMS was significantly correlated with children's WHtR.	Objective PNMS predicted central adiposity in the adolescent offspring.
		Psychological functioning (mothers)	General Health Questionnaire-28 (GHQ)		
[33]	Social Vulnerability Index (SVI)	Body composition	BIA (Tanita TBF-300A), DXA (Hologic Discovery A)	1)Prenatal stress moderated the associations of prenatal DII measures of adiposity in childhood: the magnitude of the associations of prenatal DII with was stronger among children exposed to low (β= 0.60, 95% CI:0.20-1.01) vs high (β= –0.13, 95% CI:0.93-0.66) stress in pregnancy (p= 0.03) for interaction for DII × SVI);<br><br>2)The association of prenatal DII with adiposity change did not differ between children exposed in utero to high vs low stress;<br><br>3) Associations of prenatal DII with	A proinflammatory diet in pregnancy is associated with greater adiposity accrual from childhood through adolescence, and racial and ethnic minority group and language status (the stressor) were associated with greater susceptibility to the adipogenic effects of a proinflammatory diet in pregnancy.
		Body fat	Skinfold thickness		
		Dietary Inflammation	Dietary Inflammatory Index (DII)		
		Severity of depression symptoms	Edinburgh Postpartum Depression Scale		

	change in BIA percentage body fat over time were strongest among children whose mothers had a high (β=0.55, 95%CI: 0.04-1.07) vs low (β=0.13, 95% CI:0.20-0.46) score of racial and ethnic minority subindex of SVI.	

Table S2. The characteristics of the studies participants.

Ref.	N (M/F)	Age (X± SD) [years]	BMI [kg/m²]	Study group/ Inclusion criteria	Exclusion criteria	Chronic disease or medication
[41]	36 (F:31, M:5)	24.7±0.5	22.7±0.4	Young Caucasian adults (age:18 and more) attending Harokopio University, at an under- or post-graduate level	Any systemic disease (e.g. neurological or psychiatric disorders, including clinically diagnosed anxiety disorders and depression) or treatment (e.g. sedative or antidepressant medications and relevant supplements/preparations) which could interfere with the study objectives, individuals with recent (within the past two months) significant changes in body weight (>3% of total body weight) and/or physical activity levels as well as subjects with known drug and/or alcohol use disorders were excluded from recruitment	N/A
[42]	325 (F: 165, M: 160)	7.3±0.4	16.3±2.0	First-grade pupils of public primary schools in the canton Basel-Stadt	Taking medication or supplementation	N/A
[47]	605 F	30.9±9	30.5±2.8	Women who were apparently healthy, Premenopausal, age: 18–50	History of mental disorders or using any antianxiety or antidepressant drugs or who had such diseases as	N/A

					hyper/hypothyroidism, hypertension, diabetes, early loss of ovaries, other gynecologic problems, pregnancy, lactation or any major surgery	
[21]	107 (F:55, M: 52)	27.8±7.4	26.6±5.8	Normal- and overweight patients with chronic abdominal pain	Use of daily medication for treatment of any chronic medical condition as well as any known history of gastrointestinal, pulmonological, neurological, renal, endocrinal, or gynecological pathology	N/A
[22]	241 (F:125, M:116)	F:21.5±3.0<br>M:22.4±3.1	22.0±3.1	Students over the first year of collage, age: 18 and more	N/A	N/A
[48]	57 F	Women with stress: 33.5±6.3<br>Women without stress: 29.4±7.1	N/A	Age: 20-45, BMI ranging from 25.00 to 29.99	Weight gain or loss over 3 kg in the three previous months, obesity, pre-menstrual period (10 days prior to menstruation), nutritional guidance over the six previous months, use of sweeteners, expectant mothers, lactating women, disorders such as diabetes mellitus type II, polycystic ovary syndrome, cancer, and endocrine and food disorders, hormone replacement therapy, tobacco abuse, and use of	N/A

					corticosteroids, antidepressants, or any other medication that could interfere with the appetite and emotional status	
[23]	1460 (F: 248, M: 1212)	23.5 ± 5.2	Emotional eating behavior: never: 25.5±4.5; sometimes: 25.8±3.7; often: 26.6±4.1	Active-duty Soldiers, age: 18 and more	N/A	N/A
[24]	60 (F:35, M: 25)	F:31.4±11.3, M:28.8±10.4	N/A	Age: 18-65	Metabolic disorder or cardiopulmonary disease, and taking any medication known to affect metabolism, history of claustrophobia	N/A
[25]	15 (F:7, M: 8)	13.7	N/A	Age: 10-18, BMI ≥ the 85th percentile and pubertal levels of LH	Recent oral or inhaled glucocorticoid therapy, medications for dyslipidemia or blood sugar management, a diagnosis of diabetes	N/A
[46]	4161 F Children: 4161 (F:2127, M: 2127	Mothers: 30.9±4.8 (at gestation period) Children: 9.8±3.0	22.5 (18.1-34.2) Children 16.9 (14.0-24.6)	Pregnant women	Pregnancies not leading to singleton live births	N/A
[38]	106 (F:48, M: 58)	Mothers: 35.6, fathers: 36.7	27.7±5.7	Families with at least one child aged 18 months to 5 years, resided in Wellington County, Ontario, Canada, and had a parent who could	Pregnancy or breastfeeding	N/A

				respond to questionnaires in English		
[26]	61 F	Chronic-stress:62±7, low stress: 62±6	Chronic-stress:26.4±4.9, low stress: 26.3±5.3	Postmenopausal women, age: 50-80	Chronic medical conditions (cardiovascular disease, cancer, diabetes, and autoimmune diseases), current smoking, or use of medications known to affect stress-responsive biomarkers	Antidepressant use was permitted in chronic stress group, NSAIDs: chronic-stress: 14, low-stress:4
[50]	791 (F:394, M:397)	7-8	N/A	Healthy Australian children, age: 7-8	Children from inner city schools	N/A
[27]	178 F	45.3±9.3	34.8±7.6	African American and Hispanic/Latina women aged between 25 and 60 years, able to read, speak, and write in English or Spanish	N/A	Hypertension: 32
[44]	221 (F:185, M:36)	47.8±7.7	31.1±3.1	BMI: 27–34.9, perceived psychological stress, the possibility to use computer with internet connection.	Any severe chronic illness, use of HRV-affecting or β-adrenergic blocking agent	79 participants had regular medication of those eight had a change in their medication and 13 participants started regular medication during the study period
[34]	113 (F: 53, M: 60)	12.7±1.7	N/A	Dutch-speaking Belgian children	Reported diagnosis of Cushing/Addison disease, autoimmune disease, heart disease, chronic bowel disease or acute infection, taking oral anti-inflammatory drugs, selective serotonin reuptake	N/A

					inhibitors, glucocorticosteroids or antibiotics	
[28]	F: 23	9.2±0.9	21.3±4.4	Hispanic ethnicity, female gender, Tanner breast stage 1 or 2, nonmenstruating, between the ages of 8 to 11, and meet one of the following criteria: (i) BMI ≥85th percentile (overweight or obese); or (ii) BMI <85th percentile (normal weight) with at least one parent meeting adult criteria for overweight (BMI ≥25), or diagnosed with type 2 diabetes	Diagnosis of diabetes at screening or diagnosis of a major illness or disease that could affect body composition, fat distribution, or insulin action or secretion	N/A
[35]	316	Median: 8	N/A	Belgian children, age: 5-10	N/A	N/A
[29]	5077 (F:3141, M: 1936)	N/A	N/A	Hispanic/Latino adults, age: 18-74	N/A	Asthma, chronic obstructive pulmonary disease, diabetes
[36]	1121 (F: 637, M: 484)	F:14.8±1.2 M:14.7±1.2	N/A	Data being available concerning an individual's gender, height and weight, age: 12.5-17.5	Not participating in another survey	N/A
[37]	355 (F:169, M: 186)	Median: 8	N/A	Elementary school children, age: 5-10	N/A	N/A

[30]	112: F:55, M:57	F:94.22±0.2.27, M:93.77±1.98 (in months)	F:19.09±0. 58, M: 19.43±0. 6	Latino children, age: 5-10	Chronic disease (e.g., diabetes, renal disease, liver disease), a diagnosed endocrine or hormonal cause for obesity, or medications that could affect their glucose tolerance (e.g., dilantin, corticosteroids, diuretics, β-blockers)	Glucose intolerance: 1
[51]	F:424, children: (F:210, M: 214)	Mothers: N/A Children::4.8±0.5	Children:15.6±1.7	Pregnant women prior to 20 weeks gestation	N/A	N/A
[49]	1462 (F:561. M:901	56.4±7.4	24.6±2.9	Residents in the Ansan province	N/A	N/A
[31]	US-Born African American: 183 (F:137, M:46), Afro-Caribbean imigrant: 296 (F:199, M: 97)	US-Born African American:41.4±16.9, Afro-Caribbean imigrant: 47.6±13.5	US-Born African American:28.9, Afro-Caribbean imigrant: 29.1	Non-Hispanic blacks individuals, age: 20 and more	Diagnosis of diabetes, either previously diagnosed or newly diagnosed	N/A
[32]	14044 (F: 7245, M: 6799)	29±0.03	F: 29±0.2, M: 29±0.1	Young adults during entry the study, age: 12-18	N/A	N/A
[39]	Data from 123 children between 5 (F:55, M: 56) ½ to 15 ½ year old (F:22, M:22) collected in 2-3 years intervals	Children: 5.6±0.1; 8.5±0.1; 11.6±0.1; 13.6±0.1; 15.6±0.5	Children: 5.5 year old: 15.9±1.7, 8.5 years old: 17.2±3.1; 11.5 years old: 19.6±5.2; 13.5 years old 21.6±5.3; 15.5 years old 23.7±6.4	Children of pregnant during, or conceived within 3 months of, the Quebec ice storm, age: 18 and more	N/A	N/A
[43]	318 (F:169, M:149)	7.26±3.51	16.31±2.22	First-grade pupils of public primary schools	Taking medication or supplements	N/A

[53]	1001 (F:517, M:484)	F:47.5±7.0<br>M: 47.1±7.9	F:22.8±3.2<br>M: 24.8±3.1	Teaching and non-teaching workers employed at public elementary, junior high, and senior high schools	Taking medication for hypertension, hyperuricemia, hyperlipidemia and diabetes, value for glycosylated hemoglobin ≥6.0%	N/A
[45]	Intervention: 60 (F:54, M:6), control: 64 (F:53, M:11)	Intervention:50.7±7.2<br>Control:49.5±7.4	BMI (baseline) intervention:30.8±3.0, Control:31.1±2.9; (week 36): intervention:30.2±3.2, Control:30.8±3.1	BMI: 27–34.9, perceived psychological stress, the possibility to use computer with internet connection	Diagnosed severe chronic illness (symptomatic cardiovascular disease, diabetes, severe psychiatric conditions or substance abuse), medical surgery within the past 6 months, heart attack/stroke within past 6 months, kidney disease requiring dialysis or other disabilities/illnesses affecting substantially physiological or mental health, regular use of cortisone pills, pacemaker, eating disorder (bulimia), disability pension for psychological reasons, pregnancy or breastfeeding within the past 6 months, shift work (in three shifts) or night work, psychotherapy or other psychological or mental treatment at least twice a month, and participation in other intervention studies during the present study	Users of alpha/betablockers: intervention: 7, control: 6
[52]	88 (F:45, M:43)	38 (18-45)	N/A	Healthy men and	Medication users, or people	N/A

				women, age: 18-45	with chronic disease	
[40]	66 (F:29, M:37); subsample for epigenetic analysis:31	13.60	21.1±4.7 (subsample for epigenetic analysis: 22.2±4.8)	Children of mothers whose were pregnant during the Quebec ice storm	Exposure to maternal gestational diabetes	N/A
[33]	F 1060 (children: F: 540, M:520)	Mothers: 32.6±4.6 (at gestation period); Children: 3.3±0.3 (early childhood); 7.9±08 (midchildhood); 13.2±0.9 (early adolescence)	Mothers: 24.4±4.9 (pre-pregnancy)	Mother-child dyads	Mothers: without type 1 or 2 diabetes melitus in pregnancy; children: preterm birth	N/A

N – number; F – females; M – males, SD - standard deviation; *X* – mean; BMI – body mass index; N/A – not applicable; NSAIDs - non-steroidal anti-inflammatory drugs

Table S3. Risk of bias assessment

Ref.	Study	Selection				Comparability	Exposure/ outcome			Total points
[42]	Gerber et al., 2017	*	*		*	*	**	*		7
[47]	Patel et al., 2019	*	*		*	*	**	*		7
[21]	Joseph et al., 2019	*	*	*		*	*	*		6
[22]	Hootman et al., 2018	*			*	*	**	*		6
[48]	Macedo& Diez-Garzia, 2014				*	*	**			4
[23]	Jayne et al., 2020	*	*		*	*	*	*		6
[24]	Wilson et al., 2019				*		**			3
[25]	Strait et al., 2018				*		**	*		4
[46]	Vehmeijer et al., 2019	*	*	*	*	*	*	*		7
[38]	Hruska et al., 2020				**	*	**	*		6
[26]	Aschbacher et al., 2014				*	*	**	*		5
[50]	Olive et al., 2017	*			*	*	**	*		6
[27]	Shin et al., 2016			*	**	*	**			6
[44]	F€ohr et al., 2016		*	*		*	*	*	*	6
[34]	Michels et al., 2017				*	*	**			4
[28]	Donoho et al., 2011				*	*	**	*		5
[35]	Michels et al., 2014				**	*	**	*		6
[29]	Isasi et al., 2015	*			**	*	**	*		7
[36]	De Vriendt et al., 2012	*	*		**	*	**			7
[37]	Vanaelst et al., 2014	*	*	*	**	*	**			8
[30]	Dixon et al., 2009	*			**	*	**	*		7
[51]	Wu et al., 2018	*	*	*	*	*	*	*		7
[49]	Kim et al., 2017	*			**	*	**	*		7
[31]	Tull et al., 2003	*		*	**	*	**			7
[32]	Suglia et al., 2017	*	*		**	*	**			7

[39]	Liu et al., 2016		*	*	*	*	*	*		6
[43]	Gerber et al., 2017	*		*	**	*	**			7
[53]	Yamamoto et al., 2007	*			*	*	**			5
[45]	Noerman et al., 2020		*	*		*	*	*	*	6
[52]	Jones et al., 2016				**	*	*	**		6
[40]	Cao-Lei et al., 2015		*	*	*	*	*	*		6
[33]	Monthé-Drèze et al., 2023		*	*	*	*	*	*		6
